# Association between a composite metabolic index and mortality in critically ill patients with pulmonary hypertension: a retrospective cohort study

**DOI:** 10.1038/s41598-026-56432-6

**Published:** 2026-06-03

**Authors:** Xiao-Jun Xiang, Dong-Yu Ma, Meng-Yang Liu, Li Ma, Xiao-Long Sun, Jian-Feng Li, Wen-Bo Zhang, Ping Xie

**Affiliations:** 1 School of Traditional Chinese and Western Medicine, Gansu University of Chinese Medicine, Lanzhou, 730000 China; 2 Department of Cardiovascular Medicine, Gansu Provincial Hospital, National Clinical Research Center for Cardiovascular Diseases (Branch Construction Unit), Lanzhou, 730000 China; 3 Department of Respiratory and Critical Care Medicine, Chongqing Three Gorges Medical College Affiliated Fengjie Hospital, Fengjie, 404600 Chongqing China; 4 Department of Geriatrics, Gansu Maternity and Child Health Care Hospital (Gansu Central Hospital), Lanzhou, 730000 China; 5 The First Clinical Medical College, Gansu University of Chinese Medicine, Lanzhou, 730000 China

**Keywords:** Pulmonary hypertension, Metabolic dysfunction, FBG/HDL-C, 90-day mortality, Retrospective cohort, Biomarkers, Cardiology, Diseases, Endocrinology, Medical research, Risk factors

## Abstract

Pulmonary hypertension (PH) is a heterogeneous condition with variable prognosis across World Health Organization (WHO) subtypes. The fasting blood glucose to high-density lipoprotein cholesterol (FBG/HDL-C) ratio has been suggested as a metabolic predictor of adverse outcomes in critically ill patients, but its prognostic value in PH remains unclear. This retrospective cohort included 281 ICU patients with pulmonary hypertension (PH) from MIMIC-IV (2008–2022). Patients were divided into tertiles based on ln(1 + FBG/HDL-C). The primary endpoint was 90-day all-cause mortality, which was assessed using Cox proportional hazards regression, with exploratory subgroup analyses by WHO PH groups. In fully adjusted models, patients in the highest tertile of ln(1 + FBG/HDL-C) had higher 90-day mortality than those in the lowest tertile (HR = 3.30, 95% CI: 1.82, 5.97; *p* < 0.001). Elevated risk was observed across WHO PH Groups 1, 2, and 5, with the most statistically robust and clinically interpretable association in Group 2 (HR = 2.66, 95% CI: 1.40, 5.05; *p* = 0.003). Elevated FBG/HDL-C ratio was independently associated with 90-day mortality, with the association in Group 2 being the most statistically stable and clinically interpretable.

**Supplementary Information:**

The online version contains supplementary material available at 10.1038/s41598-026-56432-6.

## Introduction

Pulmonary hypertension (PH) is defined by a resting mean pulmonary arterial pressure exceeding 20 mmHg and is characterized by increased pulmonary vascular resistance, progressive right ventricular dysfunction, and ultimately heart failure and premature death^1^. National age-standardized mortality data from the United States indicate a 34% increase in PH-related mortality between 2003 and 2020, largely driven by WHO groups 2–5 (non-PAH subtypes predominantly linked to left heart disease, lung disorders, thromboembolic disease, or multifactorial mechanisms)^2^. Most non-PAH PH subtypes, including Groups 2, 3, and 5, contribute to the majority of the global PH burden and currently lack approved disease-specific therapies^1^. In contrast, pulmonary arterial hypertension (PAH; WHO Group 1), which has multiple approved targeted pharmacological treatments, has moderate survival outcomes reported in population-based observational studies^3^. Current risk stratification strategies recommended by the European Society of Cardiology and the European Respiratory Society integrate clinical, biochemical, imaging, and hemodynamic parameters to guide management^1^. However, their reliance on specialized investigations—such as right heart catheterization and cardiopulmonary exercise testing—limits applicability in acute care and resource-limited settings, including the intensive care unit (ICU), where rapid, readily available prognostic markers are particularly needed.

Growing evidence supports that PH is not solely a pulmonary vascular disorder but a systemic metabolic–vascular disease. This paradigm, initially established in PAH^4^, has been increasingly recognized as extending beyond the pulmonary circulation to involve multiple organ systems. Within this framework, insulin resistance and associated metabolic reprogramming have emerged as central pathobiological features, influenced by complex interactions between genetic susceptibility and environmental factors^5^. In PAH, pulmonary vascular cells undergo a metabolic shift toward aerobic glycolysis (the Warburg effect), accompanied by mitochondrial fragmentation, impaired fatty acid oxidation, and redox imbalance—changes that collectively promote a hyperproliferative and apoptosis-resistant cellular phenotype^6^. Metabolomic analyses in PAH have further identified distinct circulating metabolite alterations, including polyamines, sphingolipids, and amino acids, which correlate with right ventricular dysfunction and hemodynamic severity^7^. Clinically, insulin resistance is frequently observed in PAH and precapillary PH, although its independent prognostic value remains uncertain^8^. Notably, no integrated metabolic risk score derived from routine clinical biomarkers has been validated in critically ill or heterogeneous PH populations, highlighting a critical gap between mechanistic insights and acute clinical risk stratification.

The fasting blood glucose to high-density lipoprotein cholesterol ratio (FBG/HDL-C) captures combined disturbances in glucose and lipid metabolism underlying insulin resistance. Previous work demonstrated that elevated FBG/HDL-C ratios were independently associated with short-term mortality in critically ill patients, potentially mediated through systemic inflammation and renal impairment^9^. Consistent with this observation, other insulin resistance surrogates—including TG/HDL-C and TyG-based indices—have also been linked to long-term mortality in cardiovascular and critically ill populations^10^. Recent studies have further validated the prognostic value of metabolic indices in specific ICU populations, including sepsis patients^11–13^. While indices such as the triglyceride–glucose (TyG) index and TG/HDL-C ratio are widely validated in cardiovascular and critical care populations, their applicability to pulmonary hypertension has primarily been assessed in stable outpatient cohorts with specific PH subtypes^14,15^. Whether metabolic indices, including the FBG/HDL-C ratio—which integrates acute glycemic stress with HDL-mediated vascular protection—retain prognostic value in critically ill patients with heterogeneous PH etiologies remains unclear.

Therefore, this study aimed to evaluate the association between elevated FBG/HDL-C ratio and 90-day all-cause mortality in critically ill patients with pulmonary hypertension.

## Methods

### Study design and population

This retrospective cohort study was conducted using the Medical Information Mart for Intensive Care IV (MIMIC-IV, version 3.1), a publicly available de-identified database containing detailed clinical data from ICU patients admitted to Beth Israel Deaconess Medical Center between 2008 and 2022. Data extraction, variable processing, and quality control followed previously established methods^16^. The use of the MIMIC-IV database was approved by the Institutional Review Boards of the Massachusetts Institute of Technology and Beth Israel Deaconess Medical Center, with informed consent waived due to the de-identified nature of the data. One author (X.J.X.) obtained access to the database after completing the Collaborative Institutional Training Initiative training program (certification ID: 72621916).

Pulmonary hypertension (PH) was identified using International Classification of Diseases (ICD) codes recorded in the discharge diagnosis list, including ICD-9-CM codes 416.0–416.9 and ICD-10-CM codes I27.0, I27.2, I27.20–I27.29, I27.81–I27.82, and I27.9. Eisenmenger syndrome (ICD-10-CM code I27.83) was excluded because it represents PH associated with congenital heart disease and may introduce substantial clinical heterogeneity. Patients were classified into World Health Organization (WHO) groups for pulmonary hypertension (PH) using a structured, hierarchical algorithm. Tier 1 assigned patients with direct PH subtype ICD codes to the corresponding WHO group: Group 1 (pulmonary arterial hypertension, PAH) included ICD-9-CM 416.0 and ICD-10-CM I27.0 (primary PAH) as well as I27.21 (secondary PAH); Group 2 (PH due to left heart disease, PH-LHD) included I27.22; Group 3 (PH due to lung diseases and/or hypoxia, PH-LD) included I27.23; Group 4 (chronic thromboembolic PH, CTEPH) included I27.24; and Group 5 (PH with unclear or multifactorial mechanisms, PH-U/M) included I27.29. Tier 2 was applied for patients without direct PH subtype codes. In Step 1, patients with ICD-9-CM 416.2 or ICD-10-CM I27.82 were assigned to Group 4, regardless of the presence of mild or moderate congestive heart failure (CHF) or chronic pulmonary disease (CPD), unless left heart disease was the predominant driver. In Step 2, patients with CHF comorbidity codes were assigned to Group 2, explicitly excluding those with cor pulmonale (416.9, I27.81) or isolated right heart failure (I50.81, I50.810–I50.813). Patients excluded in Step 2 were evaluated in Step 3, where the presence of CPD comorbidity codes led to assignment to Group 3. All remaining unclassified patients, including those coded as I27.9, were retained in Group 5 (unclassified/multifactorial PH).

For the primary analysis, we identified 7,655 ICU stays with pulmonary hypertension from the MIMIC-IV v3.1 database, which initially contained 94,458 ICU admissions. After excluding 3,368 patients who were not in their first hospital admission and first ICU stay, patients aged < 18 years (n = 0), 644 patients with an ICU length of stay < 24 h, 3,338 patients without HDL-C measurements (defined as the first available value within 72 h of ICU admission), and 24 patients without fasting blood glucose (FBG) measurements (defined as measurements recorded between 03:00 and 07:59 within the first 72 h of ICU admission and averaged across all available values), a total of 281 patients were included in the final analysis.

**Fig. 1:**
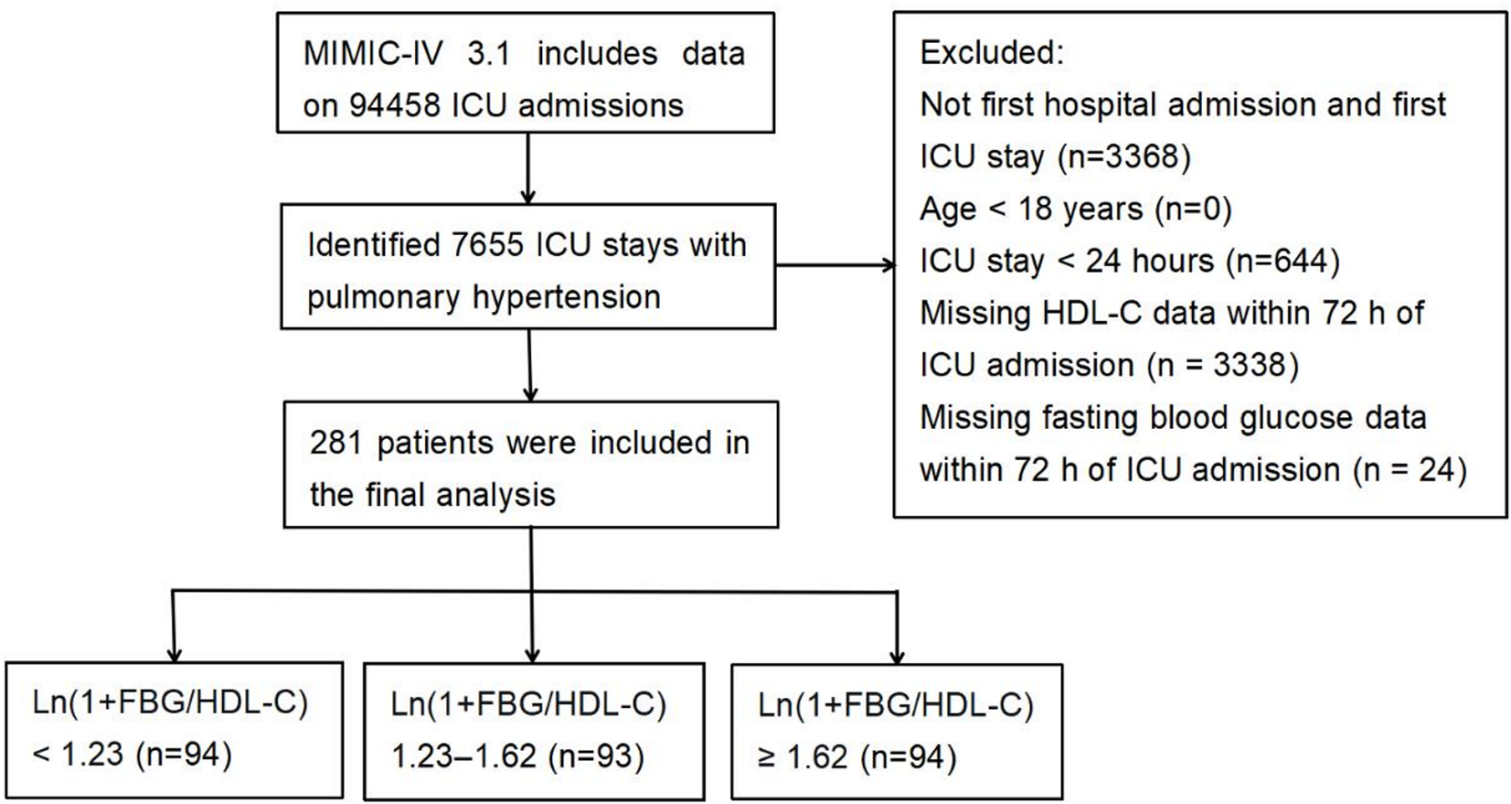
Flow diagram of participant inclusion and exclusion in the MIMIC-IV database. HDL-C, high-density lipoprotein cholesterol.

Patients were stratified into tertiles according to the ln(1 + FBG/HDL-C) ratio: T1 (< 1.23, n = 94), T2 (1.23–1.62, n = 93), and T3 (≥ 1.62, n = 94) (Fig. 1). The prespecified primary endpoint was 90-day all-cause mortality.

### Data extraction and processing

Data from the MIMIC-IV cohort were extracted using SQL in PostgreSQL. Comorbidities were defined via the Charlson Comorbidity Index (CCI) using ICD-9-CM (pre-October 2015) and ICD-10-CM codes. Diabetes, CHF, and CPD were identified per CCI (see Supplementary Table S1), while hypertension was defined separately (ICD-9-CM 4010, 4011, 4019; ICD-10-CM I10). Physiological variables, including SOFA score, and therapeutic interventions were based on the first 24-h ICU values. IMV status was determined using the MIMIC-IV ventilation concept; patients receiving any invasive ventilation within 24 h were classified as IMV.

### Missing data handling

The cohort was defined by available FBG and HDL-C, with all model covariates complete; no imputation was performed. Missing values for other baseline variables are shown in the descriptive tables.

### Calculation of ln(1 + FBG/HDL-C)

The primary exposure was the FBG/HDL-C ratio. FBG was calculated as the mean of early-morning (03:00–07:59) glucose measurements within the first 72 h of ICU admission, serving as a pragmatic fasting surrogate in critically ill patients. HDL-C was defined as the first measurement within the same window. To reduce skewness and improve model stability, the ratio was log-transformed as ln(1 + FBG/HDL-C), avoiding undefined values near zero.

### Statistical analysis

All analyses were performed using R (version 4.3.0), Python (statsmodels), and GraphPad Prism (version 10.6). A two-sided *p* < 0.05 was considered statistically significant (adjusted *p* < 0.0167 for multiple comparisons).

Baseline characteristics were compared across tertiles of ln(1 + FBG/HDL-C). Normality was assessed using the Shapiro–Wilk test. Continuous variables were expressed as mean ± SD or median (IQR) and compared using t-tests/ANOVA or Mann–Whitney U/Kruskal–Wallis tests, as appropriate. Categorical variables were presented as n (%) and compared using chi-square or Fisher’s exact tests, with Fisher’s applied for small samples. Pairwise comparisons for variables with significant overall differences were performed using post hoc tests (e.g., Dunn’s test for nonparametric data or Bonferroni-corrected t-tests/chi-square tests as appropriate). Baseline characteristics were also compared across WHO PH groups and between patients with or without HDL-C. Survival was analyzed using Kaplan–Meier curves with log-rank tests. Restricted cubic spline models (4 knots) explored potential nonlinear associations, assessed by likelihood ratio tests. Associations between ln(1 + FBG/HDL-C) and 90-day all-cause mortality were examined using Cox models, analyzing ln(1 + FBG/HDL-C) as tertiles and continuous. Three models were constructed: unadjusted; adjusted for age, sex, and weight; and further adjusted for diabetes, CHF, and SOFA score, with effect estimates per 1-unit and 1-SD increase. Multicollinearity and proportional hazards assumptions were checked using variance inflation factors and Schoenfeld residuals, with corrections as needed. Time-dependent ROC curves using IPCW^17^ evaluated predictive performance at 30, 60, and 90 days, with AUCs compared via the extended DeLong test. Pre-specified subgroup analyses and interactions were assessed using stratified Cox models and likelihood ratio tests, including separate analyses by CHF status and within each WHO PH group.

### Sensitivity analyses

First, an E-value analysis was performed to quantify the minimum strength of association that an unmeasured confounder would need to explain away the observed effects^18^. Second, the robustness of the results was further assessed by repeating the Cox proportional hazards models using alternative covariate adjustment sets, including additional adjustment for invasive mechanical ventilation (IMV) and comorbidities such as chronic pulmonary disease (CPD). Third, a sensitivity analysis was conducted by excluding patients with metastatic cancer to evaluate the potential impact of advanced malignancy on the observed associations.

## Results

### Baseline characteristics

Baseline characteristics stratified by tertiles of ln(1 + FBG/HDL-C) are shown in Table 1. Participants were categorized into T1 (< 1.23), T2 (1.23–1.62), and T3 (≥ 1.62). Higher tertiles were associated with younger age, higher body weight, and a greater proportion of males (all *p* < 0.001), while race and smoking status were similar. Diabetes prevalence increased across tertiles (*p* < 0.001), whereas hypertension and metastatic solid tumor showed no significant differences. CHF and CPD varied among groups (*p* = 0.018 and 0.024), and heart rate and systolic blood pressure differed (*p* = 0.029 and < 0.001), with no differences in respiratory rate. Significant differences were also observed in WBC, creatinine, sodium, potassium, total calcium, FBG, triglycerides, and HDL-C (all *p* < 0.05), whereas hemoglobin and platelet count were comparable. Higher tertiles exhibited greater illness severity, reflected by higher SOFA scores and a higher proportion requiring invasive mechanical ventilation (both* p* < 0.001). Pulmonary arterial hypertension–specific therapy was low and similar across groups (*p* = 0.716). Detailed post hoc pairwise comparisons are provided in Supplementary Tables S2–S3.

**Table 1:** Baseline characteristics by tertiles.

Variable	Missing, n (%)	Total (N = 281)	T1 (N = 94)	T2 (N = 93)	T3 (N = 94)	*P* value
**Demographics**						
Age, years, median (IQR)	0 (0.0)	72.8 (64.3, 83.8)	79.5 (68.6, 86.5)	74.3 (65.1, 84.8)	68.9 (61.0, 75.5)	< 0.001
Male sex, n (%)	0 (0.0)	144 (51.2)	32 (34.0)	57 (61.3)	55 (58.5)	< 0.001
Race, n (%)						0.359
White	0 (0.0)	169 (60.1)	59 (62.8)	59 (63.4)	51 (54.3)	
Other/unknown		112 (39.9)	35 (37.2)	34 (36.6)	43 (45.7)	
Weight, kg, median (IQR)	0 (0.0)	80.0 (65.8, 96.0)	73.5 (60.6, 86.4)	78.9 (67.1, 99.0)	86.0 (70.0, 100.0)	< 0.001
**Lifestyle, n (%)**						
Smoker	0 (0.0)	16 (5.7)	7 (7.4)	5 (5.4)	4 (4.3)	0.632
*Comorbidities, n (%)*						
Diabetes	0 (0.0)	98 (34.9)	20 (21.3)	32 (34.4)	46 (48.9)	< 0.001
Hypertension	0 (0.0)	101 (35.9)	39 (41.5)	35 (37.6)	27 (28.7)	0.174
CHF	0 (0.0)	191 (68.0)	54 (57.4)	71 (76.3)	66 (70.2)	0.018
CPD	0 (0.0)	155 (55.2)	51 (54.3)	61 (65.6)	43 (45.7)	0.024
Metastatic solid tumor	0 (0.0)	9 (3.2)	4 (4.3)	3 (3.2)	2 (2.1)	0.709
**Vital signs**						
Heart rate, bpm, median (IQR)	0 (0.0)	85.0 (73.0, 102.0)	81.0 (71.0, 96.0)	82.0 (72.0, 103.0)	89.0 (78.0, 104.0)	0.029
Respiratory rate, bpm, median (IQR)	0 (0.0)	20.0 (17.0, 23.0)	19.0 (16.0, 22.0)	20.0 (17.0, 22.0)	20.0 (17.0, 24.0)	0.599
Systolic blood pressure, mmHg, mean (SD)	0 (0.0)	129.4 (26.9)	135.9 (23.4)	131.6 (27.2)	120.8 (27.7)	< 0.001
**Laboratory parameters**						
Hemoglobin, g/dL, median (IQR)	2 (0.7)	11.7 (10.0, 13.2)	12.2 (10.7, 13.4)	11.4 (9.9, 13.0)	11.5 (9.3, 13.1)	0.063
White blood cell count, × 10⁹/L, median (IQR)	2 (0.7)	9.3 (7.1, 12.6)	8.0 (6.2, 10.7)	9.6 (7.3, 12.4)	10.6 (7.4, 13.8)	0.001
Platelet count, × 10⁹/L, median (IQR)	2 (0.7)	193.0 (150.0, 255.0)	191.0 (147.0, 233.0)	202.0 (167.0, 262.0)	181.0 (131.0, 262.0)	0.130
Creatinine, mg/dL, median (IQR)	3 (1.1)	1.1 (0.8, 1.6)	0.9 (0.7, 1.2)	1.2 (0.8, 1.6)	1.3 (0.9, 2.4)	< 0.001
Sodium, mmol/L, median (IQR)	3 (1.1)	139.0 (136.0, 141.0)	140.0 (137.0, 142.0)	138.0 (136.0, 141.0)	138.0 (134.0, 141.0)	0.013
Potassium, mmol/L, median (IQR)	3 (1.1)	4.2 (3.8, 4.6)	4.1 (3.7, 4.4)	4.2 (3.8, 4.5)	4.3 (3.9, 5.0)	0.024
Total calcium, mg/dL, median (IQR)	11 (3.9)	8.7 (8.3, 9.1)	8.9 (8.5, 9.3)	8.6 (8.3, 8.8)	8.6 (8.1, 8.9)	< 0.001
FBG, mg/dL, median (IQR)	0 (0.0)	123.3 (105.5, 148.0)	104.0 (93.7, 117.0)	122.0 (111.3, 141.7)	161.5 (129.5, 203.7)	< 0.001
TG, mg/dL, median (IQR)	56 (19.9)	93.0 (71.0, 118.0)	81.0 (65.0, 108.0)	94.0 (77.0, 118.0)	104.0 (83.0, 134.0)	< 0.001
HDL-C, mg/dL, median (IQR)	0 (0.0)	42.0 (31.0, 52.0)	56.0 (49.0, 68.0)	41.0 (35.0, 46.0)	28.0 (20.0, 35.0)	< 0.001
**Severity and interventions, n (%)**						
SOFA score, median (IQR)	0 (0.0)	1.0 (0.0, 2.0)	0.0 (0.0, 1.0)	1.0 (0.0, 2.0)	2.0 (0.0, 3.0)	< 0.001
IMV	0 (0.0)	73 (26.0)	12 (12.8)	25 (26.9)	36 (38.3)	< 0.001
PAH–specific therapy*	0 (0.0)	9 (3.2)	3 (3.2)	2 (2.2)	4 (4.3)	0.716

Data are presented as median (IQR) or n (%). Missing values are shown as counts (%). P values were calculated using the Kruskal–Wallis test or χ^2^ test, as appropriate. *Includes sildenafil, bosentan, epoprostenol, or tadalafil. Abbreviations: ALT, alanine aminotransferase; CHF, congestive heart failure; CPD, chronic pulmonary disease; FBG, fasting blood glucose; HDL-C, high-density lipoprotein cholesterol; IMV, invasive mechanical ventilation; SOFA, Sequential Organ Failure Assessment; TG, triglycerides; WBC, white blood cell count.

Analysis of patients with versus without HDL-C measurements revealed differences in CPD prevalence, SOFA score, and IMV use (Table S4), indicating potential selection bias, particularly given that 91.6% lacked HDL-C measurements. Baseline characteristics across WHO PH groups are summarized in Table S5. Age, sex, weight, SOFA score, and ln(FBG/HDL-C) were comparable across PH groups (all *p* > 0.05). Comorbidity patterns aligned with ICD-based grouping: CHF was universal in Group 2 (PH-LHD) and largely absent in Group 5, while CPD predominated in Group 3 (PH-LD) and was rare in Group 5. Other comorbidities, including renal disease and hypertension, varied significantly (all *p* < 0.001), reflecting distinct clinical profiles among PH subtypes. Despite these differences, 90-day all-cause mortality did not differ significantly across PH groups (*p* = 0.875). Comparisons involving Group 1 (n = 13) and Group 4 (n = 5) should be interpreted cautiously due to small sample sizes.

### Association of ln(1 + FBG/HDL-C) with all-cause mortality in PH

**Fig. 2:**
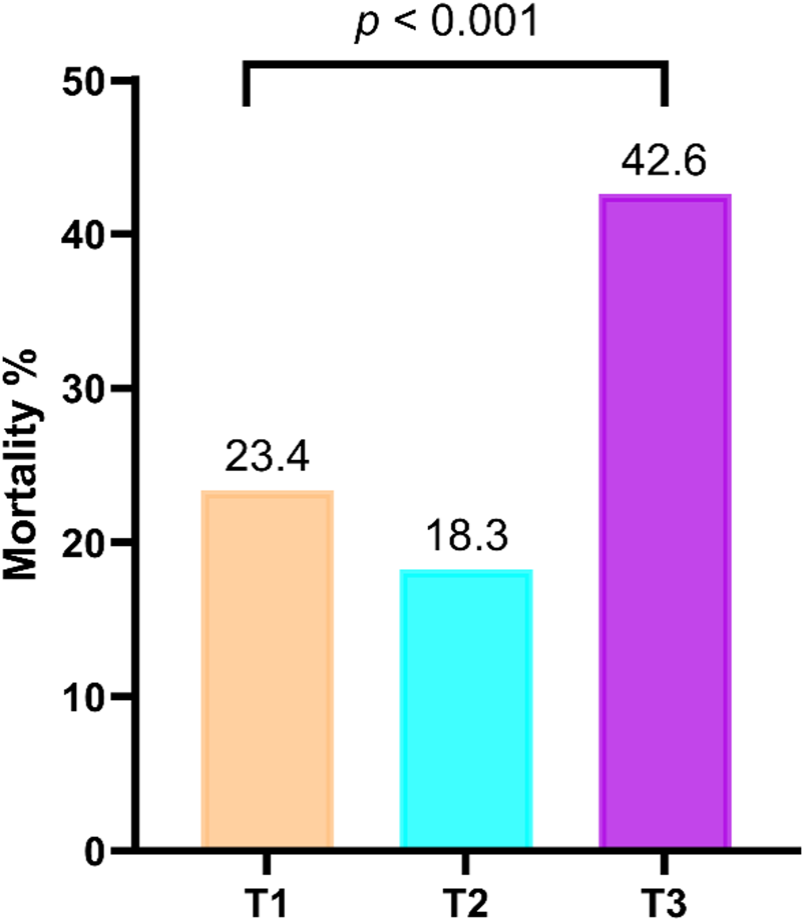
Mortality rates across T groups. The figure presents the mortality percentages for T1, T2, and T3 categories. Data are expressed as percentages (%).

A total of 79 deaths were observed, corresponding to an overall mortality rate of 28.1%. Mortality rates differed significantly across tertiles of ln(1 + FBG/HDL-C) (23.4% in T1, 18.3% in T2, and 42.6% in T3; *p* < 0.001), with a non-monotonic distribution, showing the lowest mortality in T2 and the highest in T3 (Fig. 2).

**Fig. 3:**
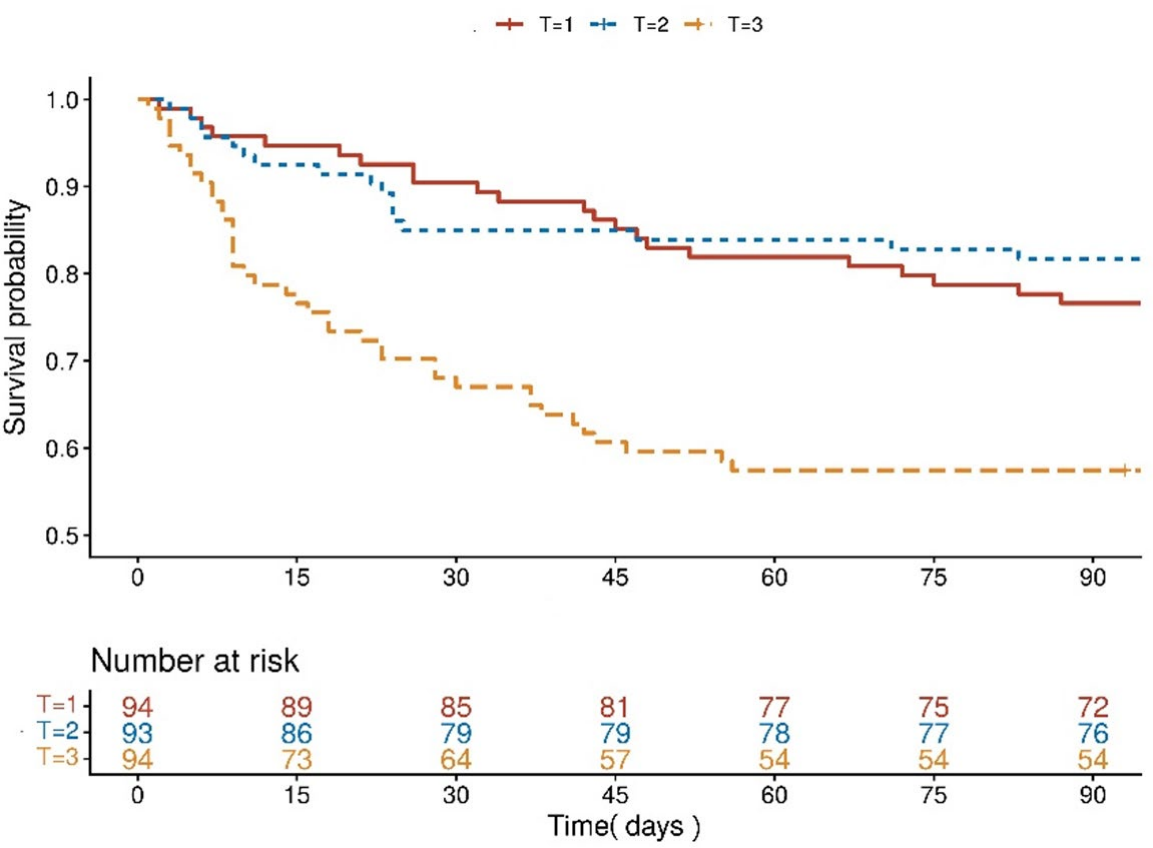
Kaplan–Meier survival curves for 90-day all-cause mortality stratified by ln(1 + FBG/HDL-C) tertiles. Differences between groups were evaluated using the log-rank test.

Kaplan–Meier survival analysis demonstrated significant differences in cumulative 90-day all-cause mortality among the three ln(1 + FBG/HDL-C) tertiles (log-rank test, *p* < 0.001) (Fig. 3).

**Fig. 4:**
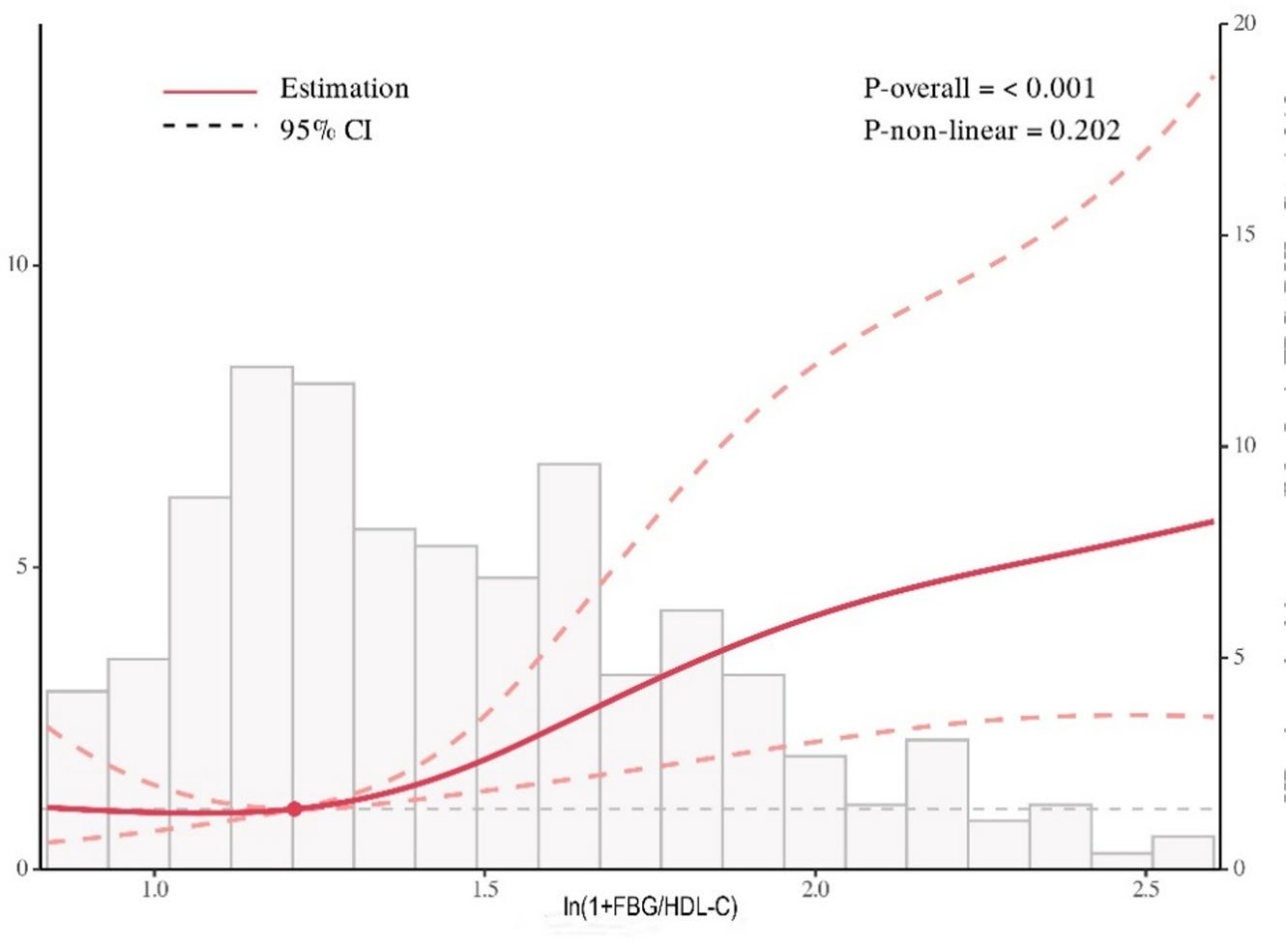
Restricted cubic spline of ln(1 + FBG/HDL-C) and 90-day mortality (n = 281). Adjusted HR (solid) with 95% CI (dashed). Significant overall association (*p* < 0.001) without evidence of nonlinearity (*p* = 0.202).

Restricted cubic spline analysis demonstrated a significant overall association between ln(1 + FBG/HDL-C) and 90-day all-cause mortality (*p* for overall < 0.001), with no evidence of a nonlinear relationship (*p* for nonlinearity = 0.202) (Fig. 4).

**Fig. 5:**
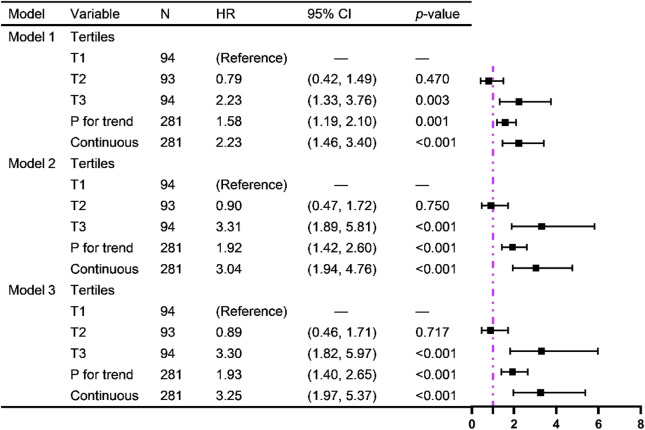
Association between ln(1 + FBG/HDL-C) and 90-day all-cause mortality among patients with pulmonary hypertension, estimated using three Cox proportional hazards models.

The results of the multivariable Cox regression analyses for 90-day all-cause mortality are shown in Fig. 5. In the fully adjusted model (Model 3), patients in the highest tertile (T3) of ln(1 + FBG/HDL-C) had a significantly higher mortality risk than those in T1 (HR = 3.30, 95% CI: 1.82, 5.97; *p* < 0.001), whereas no significant difference was observed between T2 and T1. A significant trend across tertiles was detected (*p* for trend < 0.001), primarily driven by the elevated risk in T3. When treated as a continuous variable, ln(1 + FBG/HDL-C) remained independently associated with mortality (per 1-unit increase: HR = 3.25, 95% CI: 1.97, 5.37; *p* < 0.001). Each 1-SD increase (SD = 0.46) corresponded to a 72% higher mortality risk (HR = 1.72, 95% CI: 1.37, 2.17), confirming a strong positive association. The fully adjusted model included six covariates: age, sex, weight, diabetes, congestive heart failure (CHF), and SOFA score. No evidence of multicollinearity was observed among ln(1 + FBG/HDL-C) and the included covariates (all VIFs < 5), and the proportional hazards assumption was satisfied for ln(1 + FBG/HDL-C) and all covariates.

**Fig. 6:**
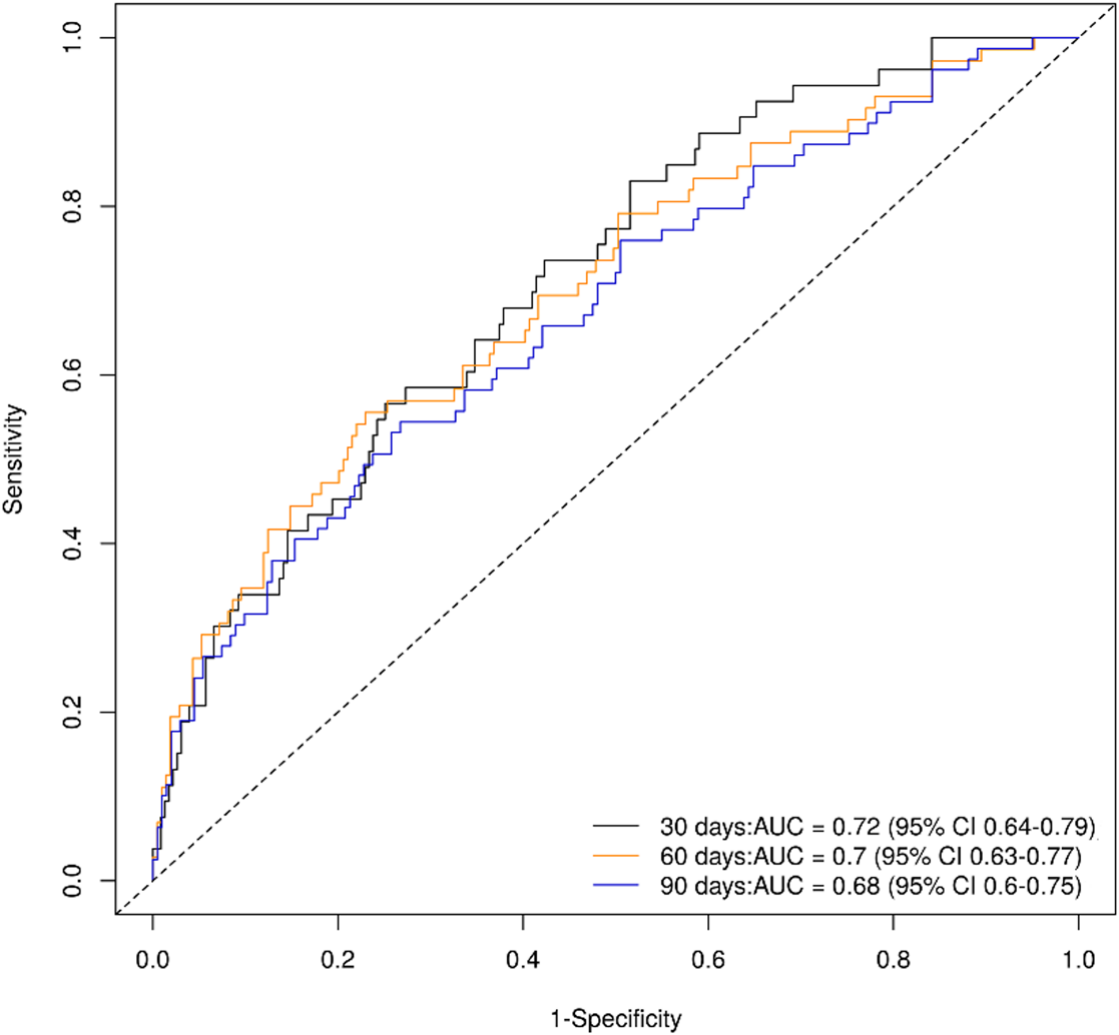
Time-dependent ROC curves illustrating the predictive ability of the ln(1 + FBG/HDL-C) for 90-day all-cause mortality.

Time-dependent ROC analysis demonstrated that the ln(1 + FBG/HDL-C) exhibited moderate predictive ability for all-cause mortality. The AUC was 0.72 (95% CI: 0.64, 0.79) at 30 days, 0.70 (95% CI: 0.63, 0.77) at 60 days, and 0.68 (95% CI: 0.60, 0.75) at 90 days (Fig. 6). The predictive performance showed a slight decreasing trend over time but remained relatively stable. Pairwise comparisons of time-dependent AUCs revealed no statistically significant differences between any two time points (all *p* > 0.05), indicating consistent discriminative performance of the ln(1 + FBG/HDL-C) during the follow-up period.

**Fig. 7:**
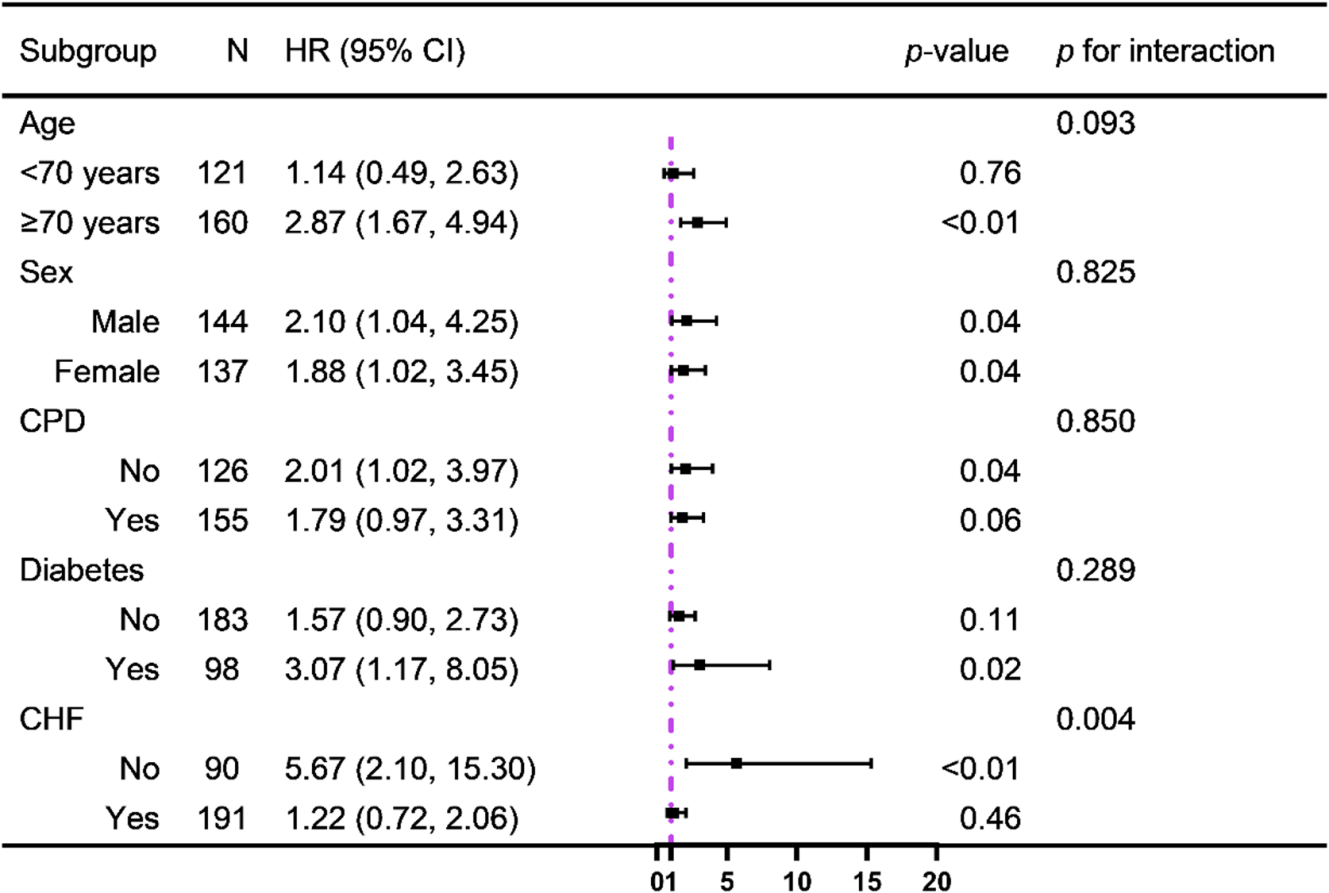
Subgroup analysis of the association between the ln(1 + FBG/HDL-C) and 90-day all-cause mortality. Models were adjusted for all covariates except the stratification variable. CHF, congestive heart failure; CPD, chronic pulmonary disease.

Subgroup analyses identified a statistically significant interaction between ln(1 + FBG/HDL-C) and CHF status for 90-day all-cause mortality (*p* for interaction = 0.004; Fig. 7). No significant interactions were observed in other prespecified subgroups, including age (*p* = 0.093), sex (*p* = 0.825), chronic pulmonary disease (*p* = 0.850), or diabetes (p = 0.289). Given the post hoc nature of this subgroup evaluation and the wide confidence intervals observed in the non-CHF stratum, these stratified analyses should be considered exploratory and hypothesis-generating.

### Analyses stratified by CHF status and WHO PH subgroups

When stratified by CHF status, ln(1 + FBG/HDL-C) was significantly associated with 90-day mortality in both patients without CHF (HR = 7.32, 95% CI: 2.74, 19.58; *p* < 0.001) and those with CHF (HR = 2.55, 95% CI: 1.40, 4.66; *p* = 0.002) in the fully adjusted model (Model 3) (Table 2).

**Table 2:** Stratified analysis by CHF status.

Model	CHF status	N	HR (95% CI)	*p*-value
Model 1	No	90	3.71 (1.72, 7.98)	0.001
	Yes	191	1.78 (1.05, 3.02)	0.034
Model 2	No	90	6.14 (2.35, 16.03)	< 0.001
	Yes	191	2.48 (1.43, 4.29)	0.001
Model 3	No	90	7.32 (2.74, 19.58)	< 0.001
	Yes	191	2.55 (1.40, 4.66)	0.002

CHF: Congestive heart failure. Model 1: univariate Cox regression. Model 2: adjusted for sex, age, and weight. Model 3: adjusted for sex, age, weight, diabetes, and SOFA score.

Subgroup analyses across WHO PH groups (Table 3) indicated that the prognostic value of ln(1 + FBG/HDL-C) was most pronounced in Group 2 (PH-LHD), where each unit increase was associated with an approximately three-fold higher 90-day mortality risk after full adjustment (Model 3: HR = 2.66, 95% CI: 1.40, 5.05, *p* = 0.003). In Group 3 (PH-LD), the association was non-significant in univariate analysis (Model 1: HR = 1.83, 95% CI: 0.61, 5.48, *p* = 0.283), suggesting limited evidence for an independent prognostic effect. In Group 5 (PH-U/M), a significant association was observed in univariate analysis (Model 1: HR = 6.60, 95% CI: 2.19, 19.89, *p* = 0.001), whereas multivariable estimates were not reported due to limited events. Estimates for the PAH (Group 1; n = 13) and CTEPH (Group 4; n = 5) subgroups are statistically unstable due to limited events and should not be overinterpreted. Overall, these findings indicate that the prognostic utility of the ln(1 + FBG/HDL-C) varies across PH etiologies, with the strongest and most consistent evidence in PH-LHD.

**Table 3:** Cox regression in PH groups

Model	Group	N	HR	95% CI	p-value
Model 1	Group 1 (PAH)	13	8.55	(1.03, 71.26)	0.047
	Group 2 (PH-LHD)	169	1.76	(0.99, 3.11)	0.053
	Group 3 (PH-LD)	49	1.83	(0.61, 5.48)	0.283
	Group 4 (CTEPH)	5	0.25	(0.00, 54.47)	0.616
	Group 5 (PH-U/M)	45	6.60	(2.19, 19.89)	0.001
Model 2	Group 2 (PH-LHD)	169	2.42	(1.33, 4.38)	0.004
Model 3	Group 2 (PH-LHD)	169	2.66	(1.40, 5.05)	0.003

Total N = 281; subgroup sample sizes reflect WHO PH classification per hierarchical ICD-based algorithm. Model 1: univariate Cox regression. Model 2: adjusted for sex, age, and weight. Model 3: adjusted for sex, age, weight, diabetes, and SOFA score. Estimates for the PAH (Group 1; n = 13) and CTEPH (Group 4; n = 5) subgroups are statistically unstable due to limited events and should not be overinterpreted. PH, pulmonary hypertension; PAH, pulmonary arterial hypertension; PH-LHD, PH due to left heart disease; PH-LD, PH due to lung diseases and/or hypoxia; CTEPH, chronic thromboembolic PH; PH-U/M, PH with unclear or multifactorial mechanisms.

### Sensitivity analyses

E-value analysis, based on the method proposed by VanderWeele and Ding, indicated that the observed associations between ln(1 + FBG/HDL-C) and 90-day all-cause mortality were relatively robust to potential unmeasured confounding. For the categorical exposure in the fully adjusted Model 3 (HR = 3.30; 95% CI: 1.82, 5.97), an unmeasured confounder would need to be associated with both the exposure and the outcome by a risk ratio of at least 6.05 to fully explain away the observed association, or 3.04 to shift the confidence interval to include the null value. For the continuous exposure (per 1-unit increase), the corresponding E-value was 5.96, while the E-value for the lower bound of the confidence interval (HR = 3.25; 95% CI: 1.97, 5.37) was3.36. In addition, sensitivity analyses using alternative covariate adjustment sets—including further adjustment for invasive mechanical ventilation (IMV) and chronic pulmonary disease (CPD)—yielded consistent results. Furthermore, after excluding patients with metastatic cancer, the association between the ln(1 + FBG/HDL-C) and 90-day all-cause mortality remained materially unchanged, suggesting that the findings were not driven by advanced malignancy.

## Discussion

In this retrospective cohort study of 281 critically ill patients with pulmonary hypertension (PH), an elevated FBG/HDL-C ratio, analyzed as ln(1 + FBG/HDL-C), was independently associated with increased 90-day all-cause mortality. Patients in the highest tertile had a significantly higher risk than those in the lowest tertile, and restricted cubic spline analysis showed a linear association without evidence of nonlinearity. Subgroup analysis revealed a significant interaction with congestive heart failure (CHF) status (*p* for interaction = 0.004). When stratified by CHF status in fully adjusted Cox models, elevated ln(1 + FBG/HDL-C) remained independently associated with 90-day mortality in both patients with CHF (HR = 2.55, 95% CI: 1.40, 4.66; *p* = 0.002) and without CHF (HR = 7.32, 95% CI: 2.74, 19.58; *p* < 0.001). The higher estimate in the non-CHF subgroup was likely driven by WHO Group 5 (PH with unclear/multifactorial mechanisms; HR = 6.60, 95% CI: 2.19, 19.89; *p* = 0.001); however, estimates for the PAH (Group 1; n = 13) and CTEPH (Group 4; n = 5) subgroups are statistically unstable due to limited events and should not be overinterpreted. Across WHO PH classifications, the association between the ln(1+FBG/HDL-C) and 90-day mortality demonstrated the greatest consistency and clinical interpretability in Group 2 (PH-LHD), where each unit increase remained independently associated with an approximately three-fold higher 90-day mortality risk after comprehensive adjustment (HR = 2.66, 95% CI: 1.40, 5.05; *p* = 0.003). Collectively, these findings indicate that the association between the FBG/HDL-C ratio and 90-day mortality varies across PH etiologies, with the strongest and most consistent evidence observed in PH-LHD. This suggests that the ratio may serve as a readily available indicator of mortality risk specifically in this population, where metabolic stress and left heart dysfunction frequently coexist^19^.

It is important to emphasize that our observational study cannot establish causality, and all interpretations should be considered exploratory rather than definitive. These findings support the view that pulmonary hypertension extends beyond a purely pulmonary vascular disorder and involves systemic metabolic dysregulation^4,5^. Specific mechanistic details, including insulin resistance and mitochondrial dysfunction, will be discussed in the context of PH-LHD below to avoid redundancy.

In particular, in PH due to left heart disease (PH-LHD), elevated left ventricular filling pressures transmit retrogradely into the pulmonary venous circulation, creating chronic mechanical stress on the pulmonary microvasculature (PCWP > 15 mmHg)^20^. This passive congestion induces endothelial dysfunction, with reduced nitric oxide (NO) bioavailability, increased oxidative stress, and pro-inflammatory activation^21^. In some patients, the condition progresses to combined post- and pre-capillary PH (CpcPH), with intrinsic vascular remodeling characterized by medial hypertrophy, intimal fibrosis, and increased pulmonary vascular resistance^22,23^. Concurrently, the underlying left heart disease, particularly heart failure with preserved ejection fraction (HFpEF)—the predominant substrate for contemporary PH-LHD—is associated with systemic metabolic-inflammatory derangements^24,25^, including insulin resistance driven by obesity and type 2 diabetes^26^, as well as hypertension. Insulin resistance has direct cardiopulmonary consequences, contributing to subclinical myocardial dysfunction^27^ and coronary microvascular impairment, which extend to the pulmonary circulation and exacerbate endothelial injury initiated by venous congestion^28^.

The FBG/HDL-C ratio emerges as a powerful integrative biomarker capturing this "dual-hit" pathophysiology. Hyperglycemia and pulmonary hypertension share common pathologic pathways involving endothelial dysfunction, inflammation, and oxidative stress^29^. At the molecular level, elevated fasting glucose reflects acute and chronic glycemic stress, promoting advanced glycation end-products (AGEs) and reactive oxygen species (ROS) overproduction^30^, leading to oxidative damage, endothelial dysfunction, and NO depletion. HDL-C normally provides vasoprotective effects, but in systemic metabolic disease it becomes dysfunctional or pro-inflammatory, with reduced paraoxonase-1 activity, MPO-mediated modification, and impaired endothelial NO synthase stimulation^31^. In PH-LHD, this loss of HDL protection amplifies vascular injury and remodeling^32^, while dysregulated fatty acid metabolism in pericardial adipose tissue further underscores the systemic metabolic perturbation driving disease progression^33^. Thus, a high FBG/HDL-C ratio signifies amplified metabolic and oxidative stress with impaired vascular defense, providing a pragmatic clinical surrogate for disease severity. Our previous study has established the prognostic value of this ratio in critically ill patients^9^, and our current data extend this observation to the PH-LHD population, where a high ratio was strongly associated with 90-day mortality. In our cohort, time-dependent ROC analysis showed that the FBG/HDL-C ratio had moderate discriminative ability for 90-day all-cause mortality, with area under the curve (AUC) values of 0.72 (95% CI: 0.64, 0.79) at 30 days, 0.70 (95% CI: 0.63, 0.77) at 60 days, and 0.68 (95% CI: 0.60, 0.75) at 90 days. Time-dependent ROC analysis indicated moderate but consistent predictive performance, supporting its potential use as part of an integrated prognostic framework alongside clinical and physiological parameters.

Therapeutically, interventions targeting metabolic health, such as SGLT2 inhibitors, may improve insulin sensitivity, reduce inflammation, and ameliorate the pulmonary vascular phenotype, highlighting both prognostic and potential therapeutic relevance of the FBG/HDL-C ratio in this population^34^. Emerging evidence also suggests that FABP4 inhibition may attenuate right ventricular fibrosis in metabolic syndrome-related PH-LHD, pointing to novel therapeutic targets beyond traditional hemodynamic management^35^. Thus, the FBG/HDL-C ratio may serve as a readily available indicator of mortality risk in PH-LHD, while simultaneously highlighting the potential relevance of metabolic dysregulation as a target for future therapeutic strategies.

Several limitations should be acknowledged. First, the very high proportion of patients lacking HDL-C measurements (91.6%) may have introduced substantial selection bias, as those with available lipid data were likely enriched for metabolic dysfunction and differed systematically from the excluded population. A bias assessment comparing patients with and without HDL-C measurements (Supplementary Table S4) showed generally similar baseline characteristics for most variables, although differences were noted in CPD, SOFA score, and IMV usage. Second, PH diagnoses were based on ICD codes, which—while structured into WHO groups using a hierarchical algorithm—may still introduce misclassification and heterogeneity; although patients were assigned to WHO Groups 1–5 based on ICD-9/10 codes and comorbidity rules, some subgroup sample sizes were small, limiting statistical power and the interpretability of subgroup-specific findings. Third, fasting blood glucose values were approximated using early-morning ICU measurements, which may not fully represent standardized fasting conditions. Fourth, HDL-C concentration, while routinely measured, does not capture HDL functionality, which may be altered in critical illness or metabolic disease. Fifth, the retrospective observational design precludes causal inference, and observed associations should be considered exploratory rather than definitive. Finally, the overall sample size, particularly in subgroups, and the fact that this is a single-center study, limit the stability of multivariable models and interaction analyses, and the predictive performance of the FBG/HDL-C ratio should be interpreted cautiously within these constraints.

Despite these limitations, this study provides novel evidence linking the FBG/HDL-C ratio to 90-day mortality in critically ill patients with pulmonary hypertension. The association was strongest and most clinically interpretable in Group 2 (PH-LHD), where each unit increase corresponded to a three-fold higher risk, highlighting its relevance in the predominant PH phenotype. As a simple, readily available biomarker integrating glucose and lipid metabolism, the FBG/HDL-C ratio may offer additional prognostic insight in acute care settings. Future prospective, multicenter studies with detailed hemodynamic assessment are needed to validate these findings and further clarify the role of metabolic dysregulation in PH prognosis.

## Conclusion

In this retrospective cohort of 281 critically ill patients with pulmonary hypertension, an elevated FBG/HDL-C ratio was independently associated with increased 90-day all-cause mortality, with the association in PH due to left heart disease (PH-LHD, WHO Group 2) being the most statistically robust and clinically interpretable.

## Supplementary Information

Below is the link to the electronic supplementary material.

Supplementary Material 1

## Data Availability

The data used in this study were obtained from the Medical Information Mart for Intensive Care IV (MIMIC-IV, version 3.1), a publicly accessible critical care database hosted on PhysioNet (https://physionet.org/). Access to the database was granted after completion of the required credentialing process, including training in human subjects research through the Collaborative Institutional Training Initiative (CITI) Program.

## Abbreviations

AUC: Area under the curve
CCI: Charlson comorbidity index
CHF: Congestive heart failure
CPD: Chronic pulmonary disease
CpcPH: Combined post- and pre-capillary pulmonary hypertension
FBG: Fasting blood glucose
HDL-C: High-density lipoprotein cholesterol
HFpEF: Heart failure with preserved ejection fraction
ICD: International Classification of Diseases
IMV: Invasive mechanical ventilation
IQR: Interquartile range
KM: Kaplan–Meier
NO: Nitric oxide
PAH: Pulmonary arterial hypertension (WHO Group 1)
PCWP: Pulmonary capillary wedge pressure
PH: Pulmonary hypertension
PH-LHD: Pulmonary hypertension due to left heart disease (WHO Group 2)
PH-LD: Pulmonary hypertension due to lung diseases/hypoxia (WHO Group 3)
CTEPH: Chronic thromboembolic pulmonary hypertension (WHO Group 4)
PH-U/M: Pulmonary hypertension with unclear or multifactorial mechanisms (WHO Group 5)
RCS: Restricted cubic spline
ROC: Receiver operating characteristic
SD: Standard deviation
SOFA: Sequential Organ Failure Assessment
TG: Triglycerides
WBC: White blood cell count

## References

[1] Humbert, M. et al. 2022 ESC/ERS guidelines for the diagnosis and treatment of pulmonary hypertension. *Eur. Respir. J.* **61**(1), 2200879. 10.1183/13993003.00879-2022 (2023). 36028254 10.1183/13993003.00879-2022

[2] Singh, H. et al. Pulmonary hypertension associated mortality in the United States from 2003 to 2020: An observational analysis of time trends and disparities. *J. Thorac. Dis.* **15**(6), 3256–3272. 10.21037/jtd-22-1468 (2023). 37426148 10.21037/jtd-22-1468 PMC10323561

[3] Reinders, S., Didden, E. & Ong, R. Survival, morbidity, and quality of life in pulmonary arterial hypertension patients: A systematic review of outcomes reported by population-based observational studies. *Respir. Res.* **25**(1), 373. 10.1186/s12931-024-02994-w (2024). 39415261 10.1186/s12931-024-02994-w PMC11481430

[4] Sutendra, G. & Michelakis, E. D. The metabolic basis of pulmonary arterial hypertension. *Cell Metab.* **19**(4), 558–573. 10.1016/j.cmet.2014.01.004 (2014). 24508506 10.1016/j.cmet.2014.01.004

[5] Zanotto, T. M., Gonçalves, A. E. D. S. & Saad, M. J. A. Pulmonary hypertension and insulin resistance: A mechanistic overview. *Front. Endocrinol. (Lausanne)* **14**, 1283233. 10.3389/fendo.2023.1283233 (2024). 38239990 10.3389/fendo.2023.1283233 PMC10794542

[6] Zhang, W. et al. Mitochondrial dysfunction in pulmonary arterial hypertension. *Front. Physiol.* **13**, 1079989. 10.3389/fphys.2022.1079989 (2022). 36589421 10.3389/fphys.2022.1079989 PMC9795033

[7] Pi, H. et al. Metabolomic signatures associated with pulmonary arterial hypertension outcomes. *Circ. Res.* **132**(3), 254–266. 10.1161/CIRCRESAHA.122.321923 (2023). 36597887 10.1161/CIRCRESAHA.122.321923 PMC9904878

[8] Zare, E. et al. Prognostic significance of insulin resistance in pulmonary hypertension. *ESC Heart Fail.* **9**(1), 318–326. 10.1002/ehf2.13752 (2022). 34904389 10.1002/ehf2.13752 PMC8788000

[9] Xiang, X. et al. Prognostic value of the FBG/HDL-C ratio for 28-day all-cause mortality in critically ill patients: A retrospective cohort study with external validation. *Eur. J. Med. Res.*10.1186/s40001-026-04141-1 (2026). 41764556 10.1186/s40001-026-04141-1

[10] Zhou, Z. et al. Comparative study on the predictive value of TG/HDL-C, TyG and TyG-BMI indices for 5-year mortality in critically ill patients with chronic heart failure: A retrospective study. *Cardiovasc. Diabetol.* **23**(1), 213. 10.1186/s12933-024-02308-w (2024). 38902757 10.1186/s12933-024-02308-w PMC11191322

[11] Lou, J. et al. A retrospective study utilized MIMIC-IV database to explore the potential association between triglyceride-glucose index and mortality in critically ill patients with sepsis. *Sci. Rep.* **14**(1), 24081. 10.1038/s41598-024-75050-8 (2024). 39402158 10.1038/s41598-024-75050-8 PMC11473526

[12] Zheng, R. et al. Association between triglyceride-glucose index and in-hospital mortality in critically ill patients with sepsis: Analysis of the MIMIC-IV database. *Cardiovasc. Diabetol.* **22**(1), 307. 10.1186/s12933-023-02041-w (2023). 37940931 10.1186/s12933-023-02041-w PMC10634031

[13] Xu, H. et al. Association of TyG index with mortality at 28 days in sepsis patients in intensive care from MIMIC IV database. *Sci. Rep.* **15**(1), 2344. 10.1038/s41598-025-86746-w (2025). 39833386 10.1038/s41598-025-86746-w PMC11747252

[14] Gao, L. et al. Association of insulin resistance surrogates with disease severity and adverse outcomes in chronic thromboembolic pulmonary hypertension: A multicenter cohort study. *Cardiovasc. Diabetol.* **24**(1), 82. 10.1186/s12933-025-02630-x (2025). 39972493 10.1186/s12933-025-02630-x PMC11841354

[15] Zhang, S. et al. Association of non-insulin-based insulin resistance indices with disease severity and adverse outcome in idiopathic pulmonary arterial hypertension: A multi-center cohort study. *Cardiovasc. Diabetol.* **23**(1), 154. 10.1186/s12933-024-02236-9 (2024). 38702735 10.1186/s12933-024-02236-9 PMC11069206

[16] Johnson, A. E. W. et al. MIMIC-IV, a freely accessible electronic health record dataset. *Sci. Data.* **10**(1), 1. 10.1038/s41597-022-01899-x (2023). 36596836 10.1038/s41597-022-01899-x PMC9810617

[17] Heagerty, P. J. & Zheng, Y. Survival model predictive accuracy and ROC curves. *Biometrics* **61**(1), 92–105. 10.1111/j.0006-341X.2005.030814.x (2005). 15737082 10.1111/j.0006-341X.2005.030814.x

[18] VanderWeele, T. J. & Ding, P. Sensitivity analysis in observational research: Introducing the E-value. *Ann. Intern. Med.* **167**(4), 268–274. 10.7326/M16-2607 (2017). 28693043 10.7326/M16-2607

[19] Weng, J. et al. High triglyceride-to-high-density lipoprotein cholesterol ratio predicts poor prognosis in new-onset heart failure: A retrospective study. *BMC Cardiovasc. Disord.* **25**(1), 251. 10.1186/s12872-025-04706-8 (2025). 40175907 10.1186/s12872-025-04706-8 PMC11963554

[20] Al-Omary, M. S., Sugito, S., Boyle, A. J., Sverdlov, A. L. & Collins, N. J. Pulmonary hypertension due to left heart disease: Diagnosis, pathophysiology, and therapy. *Hypertension* **75**(6), 1397–1408. 10.1161/HYPERTENSIONAHA.119.14330 (2020). 32336230 10.1161/HYPERTENSIONAHA.119.14330

[21] De Luca, M. et al. Endothelial dysfunction and heart failure with preserved ejection fraction: An updated review of the literature. *Life* **14**(1), 30. 10.3390/life14010030 (2023). 38255646 10.3390/life14010030 PMC10817572

[22] Ltaief, Z., Yerly, P. & Liaudet, L. Pulmonary hypertension in left heart diseases: pathophysiology, hemodynamic assessment and therapeutic management. *Int. J. Mol. Sci.* **24**(12), 9971. 10.3390/ijms24129971 (2023). 37373119 10.3390/ijms24129971 PMC10298585

[23] Fernández, A. I. et al. The biological bases of group 2 pulmonary hypertension. *Int. J. Mol. Sci.* **20**(23), 5884. 10.3390/ijms20235884 (2019). 31771195 10.3390/ijms20235884 PMC6928720

[24] Shah, S. J. et al. Research priorities for heart failure with preserved ejection fraction: National Heart, Lung, and Blood Institute Working Group Summary. *Circulation* **141**(12), 1001–1026. 10.1161/CIRCULATIONAHA.119.041886 (2020). 32202936 10.1161/CIRCULATIONAHA.119.041886 PMC7101072

[25] Schiattarella, G. G., Rodolico, D. & Hill, J. A. Metabolic inflammation in heart failure with preserved ejection fraction. *Cardiovasc. Res.* **117**(2), 423–434. 10.1093/cvr/cvaa217 (2021). 32666082 10.1093/cvr/cvaa217 PMC8599724

[26] Stoicescu, L., Crişan, D., Morgovan, C., Avram, L. & Ghibu, S. Heart failure with preserved ejection fraction: the pathophysiological mechanisms behind the clinical phenotypes and the therapeutic approach. *Int. J. Mol. Sci.* **25**(2), 794. 10.3390/ijms25020794 (2024). 38255869 10.3390/ijms25020794 PMC10815792

[27] Gudenkauf, B. et al. Insulin resistance is associated with subclinical myocardial dysfunction and reduced functional capacity in heart failure with preserved ejection fraction. *J. Cardiol.* **83**(2), 100–104. 10.1016/j.jjcc.2023.06.008 (2024). 37364818 10.1016/j.jjcc.2023.06.008 PMC12973288

[28] Cuijpers, I. et al. Microvascular and lymphatic dysfunction in HFpEF and its associated comorbidities. *Basic Res. Cardiol.* **115**(4), 39. 10.1007/s00395-020-0798-y (2020). 32451732 10.1007/s00395-020-0798-y PMC7248044

[29] Bruck, O. & Pandit, L. M. Pulmonary hypertension and hyperglycemia: not a sweet combination. *Diagnostics (Basel).* **14**(11), 1119. 10.3390/diagnostics14111119 (2024). 38893645 10.3390/diagnostics14111119 PMC11171670

[30] Li, Y. et al. Diabetic vascular diseases: molecular mechanisms and therapeutic strategies. *Signal Transduct. Target Ther.* **8**, 152. 10.1038/s41392-023-01400-z (2023). 37037849 10.1038/s41392-023-01400-z PMC10086073

[31] Chapman, M. J. HDL functionality in type 1 and type 2 diabetes: new insights. *Curr. Opin Endocrinol. Diabetes Obes.* **29**(2), 112–123. 10.1097/MED.0000000000000705 (2022). 34980868 10.1097/MED.0000000000000705 PMC8915990

[32] Ranchoux, B. et al. Metabolic syndrome exacerbates pulmonary hypertension due to left heart disease. *Circ. Res.* **125**(4), 449–466. 10.1161/CIRCRESAHA.118.314555 (2019). 31154939 10.1161/CIRCRESAHA.118.314555

[33] Qiu, H. et al. Dysregulated fatty acid metabolism in pericardiac adipose tissue of pulmonary hypertension due to left heart disease mice. *FASEB J.* **39**(3), e70355. 10.1096/fj.202402842R (2025). 39932146 10.1096/fj.202402842R PMC11812284

[34] King, N. E. & Brittain, E. Emerging therapies: The potential roles of SGLT2 inhibitors, GLP-1 agonists, and ARNI therapy for pulmonary hypertension. *Pulm. Circ.* **12**(1), e12028. 10.1002/pul2.12028 (2022). 35506082 10.1002/pul2.12028 PMC9052991

[35] Qiu, H. et al. FABP4 inhibitor improves right ventricular fibrosis in metabolic syndrome-related pulmonary hypertension due to left heart disease in mice. *FASEB J.* **39**(14), e70833. 10.1096/fj.202500532RR (2025). 40667722 10.1096/fj.202500532RR

