# Supplementary material for: Association between a composite metabolic index and mortality in critically ill patients with pulmonary hypertension: a retrospective cohort study
Source: Sci Rep. 2026 Jun 3;16:25223. doi: 10.1038/s41598-026-56432-6 (PMC13469665; doi:10.1038/s41598-026-56432-6)
Supplement: Supplementary file 1 [file 41598_2026_56432_MOESM1_ESM.docx]

Table S1. ICD codes used to define CCI comorbidities

| Comorbidity | ICD-9-CM Codes | ICD-10-CM Codes |
| --- | --- | --- |
| Diabetes (without complications) | 250.00–250.33 | E10.9, E11.9, E13.9, E14.9 |
| Diabetes (with complications) | 250.40–250.93 | E10.0, E10.1, E10.2–E10.8, E11.0, E11.1, E11.2–E11.8, E13.0, E13.1, E13.2–E13.8, E14.0–E14.8 |
| CHF | 398.91, 402.01, 402.11,  402.91, 404.01, 404.03,  404.11, 404.13, 404.91,  404.93, 428.0–428.9 | I09.81, I11.0, I13.0, I13.2,  I50.0–I50.9 |
| CPD | 490–496, 500–505, 506.4,  508.1, 508.8, 416.8, 416.9 | J40–J47, J60–J67, J68.4, J70.1, J70.3, J70.8, I27.8, I27.9 |

ICD, International Classification of Diseases; CCI, Charlson Comorbidity Index; CHF, congestive heart failure; CPD, chronic pulmonary disease.

Table S2. Multiple comparisons of clinical indicators among three groups

| Variable | Comparison | Statistic | Adjusted *p* value | Notes |
| --- | --- | --- | --- | --- |
| Age | T1 vs T2 | Mean rank diff = 12.45 | 0.8844 | Dunn’s test |
|  | T1 vs T3 | Mean rank diff = 47.44 | 0.0002** |  |
|  | T2 vs T3 | Mean rank diff = 34.98 | 0.0097** |  |
| Weight | T1 vs T2 | Mean rank diff = –27.27 | 0.0653 | Dunn’s test |
|  | T1 vs T3 | Mean rank diff = –48.19 | 0.0001** |  |
|  | T2 vs T3 | Mean rank diff = –20.92 | 0.2352 |  |
| Heart rate | T1 vs T2 | Mean rank diff = –6.766 | >0.9999 | Dunn’s test |
|  | T1 vs T3 | Mean rank diff = –30.07 | 0.0335* |  |
|  | T2 vs T3 | Mean rank diff = –23.30 | 0.1496 |  |
| SBP | T1 vs T2 | Mean diff = 4.270 | 0.5085 | Tukey’s test |
|  | T1 vs T3 | Mean diff = 15.02 | 0.0003** |  |
|  | T2 vs T3 | Mean diff = 10.75 | 0.0153** |  |
| WBC | T1 vs T2 | Mean rank diff = –29.59 | 0.0378* | Dunn’s test |
|  | T1 vs T3 | Mean rank diff = –41.85 | 0.0012** |  |
|  | T2 vs T3 | Mean rank diff = –12.26 | 0.9002 |  |
| Creatinine | T1 vs T2 | Mean rank diff = –39.13 | 0.0027** | Dunn’s test |
|  | T1 vs T3 | Mean rank diff = –60.91 | <0.0001** |  |
|  | T2 vs T3 | Mean rank diff = –21.78 | 0.1943 |  |
| Sodium | T1 vs T2 | Mean rank diff = 20.97 | 0.2256 | Dunn’s test |
|  | T1 vs T3 | Mean rank diff = 34.40 | 0.0103** |  |
|  | T2 vs T3 | Mean rank diff = 13.43 | 0.7634 |  |
| Potassium | T1 vs T2 | Mean rank diff = –13.63 | 0.7448 | Dunn’s test |
|  | T1 vs T3 | Mean rank diff = –31.98 | 0.0198* |  |
|  | T2 vs T3 | Mean rank diff = –18.36 | 0.3598 |  |
| Total calcium | T1 vs T2 | Mean rank diff = 38.55 | 0.0028** | Dunn’s test |
|  | T1 vs T3 | Mean rank diff = 38.82 | 0.0024** |  |
|  | T2 vs T3 | Mean rank diff = 0.2781 | >0.9999 |  |
| FBG | T1 vs T2 | Mean rank diff = –60.31 | <0.0001** | Dunn’s test |
|  | T1 vs T3 | Mean rank diff = –125.7 | <0.0001** |  |
|  | T2 vs T3 | Mean rank diff = –65.40 | <0.0001** |  |
| TG | T1 vs T2 | Mean rank diff = –22.52 | 0.0990 | Dunn’s test |
|  | T1 vs T3 | Mean rank diff = –38.38 | 0.0007** |  |
|  | T2 vs T3 | Mean rank diff = –15.86 | 0.4529 |  |
| HDL-C | T1 vs T2 | Mean rank diff = 81.17 | <0.0001** | Dunn’s test |
|  | T1 vs T3 | Mean rank diff = 155.2 | <0.0001** |  |
|  | T2 vs T3 | Mean rank diff = 74.02 | <0.0001** |  |
| SOFA | T1 vs T2 | Mean rank diff = –8.543 | >0.9999 | Dunn’s test |
|  | T1 vs T3 | Mean rank diff = –42.41 | 0.0005** |  |
|  | T2 vs T3 | Mean rank diff = –33.87 | 0.0078** |  |

Data are presented as mean differences or mean rank differences. Pairwise comparisons were conducted using post hoc tests following overall group comparisons (Kruskal–Wallis test with Dunn’s test or one-way ANOVA with Bonferroni correction, as appropriate). Adjusted P values for multiple comparisons are reported. A two-sided P < 0.05 was considered statistically significant (*), and an adjusted P < 0.0167 was considered statistically significant after Bonferroni correction (**). SBP, systolic blood pressure; WBC, white blood cell count; FBG, fasting blood glucose; TG, triglycerides; HDL-C, high-density lipoprotein cholesterol; SOFA, Sequential Organ Failure Assessment.

Table S3. Binary variables: baseline characteristics and tertile comparisons

| Variable | Comparison | Δ† | Adjusted *P*‡ |
| --- | --- | --- | --- |
| Sex (male) | T1 vs T2 | +27.3% | <0.001** |
|  | T1 vs T3 | +23.3% | 0.001** |
|  | T2 vs T3 | –4.0% | — |
| Diabetes | T1 vs T2 | +13.1% | 0.045* |
|  | T1 vs T3 | +27.6% | <0.001** |
|  | T2 vs T3 | +14.5% | 0.044* |
| CHF | T1 vs T2 | +18.9% | 0.006** |
|  | T1 vs T3 | +12.8% | — |
|  | T2 vs T3 | –6.1% | — |
| CPD | T1 vs T2 | +11.3% | — |
|  | T1 vs T3 | –8.5% | — |
|  | T2 vs T3 | –19.9% | 0.006** |
| IMV | T1 vs T2 | +14.1% | 0.015* |
|  | T1 vs T3 | +25.9% | <0.001** |
|  | T2 vs T3 | +11.8% | — |

Data are presented as percentage differences (Δ). †Δ = percentage in higher tertile − percentage in lower tertile. ‡Adjusted *P* values were obtained using χ² tests with Bonferroni correction (α = 0.0167); “—” indicates adjusted *P* > 0.05. A two-sided *P* < 0.05 was considered statistically significant (*), and an adjusted *p* < 0.0167 was considered statistically significant after Bonferroni correction (**).CHF, congestive heart failure; CPD, chronic pulmonary disease; IMV, invasive mechanical ventilation.

Table S4. Baseline characteristics of patients with and without HDL-C measurements

| Variable | Total (N = 3643) | HDL-C not measured (N = 3338) | HDL-C measured  (N = 305) | SMD (95% CI) | *p* value |
| --- | --- | --- | --- | --- | --- |
| Age, years, median [IQR] | 73.4 (62.7, 82.6) | 73.4 (62.6, 82.4) | 73.8 (64.6, 84.1) | 0.1 (-0.1, 0.2) | 0.296 |
| Sex, n (%) |  |  |  | 0.1 (-0.1, 0.2) | 0.376 |
| Male | 1763 (48.4) | 1608 (48.2) | 155 (50.8) |  |  |
| Female | 1880 (51.6) | 1730 (51.8) | 150 (49.2) |  |  |
| Weight, kg, median [IQR] | 78.3 (65.1, 96.0) | 78.0 (65.1, 96.0) | 79.0 (65.5, 95.8) | 0.0 (-0.1, 0.1) | 0.928 |
| Hypertension |  |  |  | 0.1 (-0.0, 0.2) | 0.209 |
| No | 2447 (67.2) | 2252 (67.5) | 195 (63.9) |  |  |
| Yes | 1196 (32.8) | 1086 (32.5) | 110 (36.1) |  |  |
| Diabetes |  |  |  | 0.0 (-0.1, 0.2) | 0.558 |
| No | 2432 (66.8) | 2233 (66.9) | 199 (65.2) |  |  |
| Yes | 1211 (33.2) | 1105 (33.1) | 106 (34.8) |  |  |
| CHF |  |  |  |  | 0.615 |
| No | 1242 (34.1) | 1142 (34.2) | 100 (32.8) |  |  |
| Yes | 2401 (65.9) | 2196 (65.8) | 205 (67.2) |  |  |
| Renal disease |  |  |  | 0.0 (-0.1, 0.2) | 0.427 |
| No | 2470 (67.8) | 2257 (67.6) | 213 (69.8) |  |  |
| Yes | 1173 (32.2) | 1081 (32.4) | 92 (30.2) |  |  |
| CPD |  |  |  | 0.2 (0.1, 0.4) | <0.001 |
| No | 1296 (35.6) | 1155 (34.6) | 141 (46.2) |  |  |
| Yes | 2347 (64.4) | 2183 (65.4) | 164 (53.8) |  |  |
| Metastatic solid tumor |  |  |  | 0.1 (-0.1, 0.2) | 0.415 |
| No | 3491 (95.8) | 3196 (95.7) | 295 (96.7) |  |  |
| Yes | 152 (4.2) | 142 (4.3) | 10 (3.3) |  |  |
| SOFA score, median [IQR] | 1.0 (0.0, 3.0) | 1.0 (0.0, 4.0) | 1.0 (0.0, 2.0) | 0.5 (0.4, 0.6) | <0.001 |
| IMV, n (%) |  |  |  | 0.3 (0.2, 0.4) | <0.001 |
| No | 2137 (58.7) | 1918 (57.5) | 219 (71.8) |  |  |
| Yes | 1506 (41.3) | 1420 (42.5) | 86 (28.2) |  |  |

Data are median [IQR] or n (%). P-values: Mann–Whitney U test (continuous) or χ² test (categorical); two-sided P < 0.05 significant. SMD, standardized mean difference (95% CI); |SMD| < 0.1 indicates adequate balance. Abbreviations: HDL-C, high-density lipoprotein cholesterol; CHF, congestive heart failure; CPD, chronic pulmonary disease; SOFA, Sequential Organ Failure Assessment; IMV, invasive mechanical ventilation.

**Table S5. Baseline characteristics of patients across PH groups**

| Variable | Overall (N=281) | Group 1 (n=13) | Group 2 (n=169) | Group 3 (n=49) | Group 4 (n=5) | Group 5 (n=45) | *p*-value |
| --- | --- | --- | --- | --- | --- | --- | --- |
| **Age, median (IQR), years** | 72.82 (64.30, 83.83) | 74.68 (66.45, 84.99) | 72.81 (64.64, 83.83) | 77.45 (68.31, 85.80) | 73.82 (72.82, 74.39) | 69.30 (55.32, 82.69) | 0.281 |
| Sex, n (%) |  |  |  |  |  |  | 0.466 |
| Male | 144 (51.2) | 5 (38.5) | 91 (53.8) | 23 (46.9) | 1 (20.0) | 24 (53.3) |  |
| Female | 137 (48.8) | 8 (61.5) | 78 (46.2) | 26 (53.1) | 4 (80.0) | 21 (46.7) |  |
| **Weight,**  **median**  **(IQR), kg** | 80.00 (65.80, 96.00) | 83.20 (76.40, 90.20) | 80.00 (66.00, 98.10) | 77.20 (60.60, 89.00) | 74.70 (63.00, 77.70) | 82.00 (68.30, 94.20) | 0.348 |
| **SOFA,**  **median (IQR)** | 1.00  (0.00, 2.00) | 1.00 (0.00, 2.00) | 1.00  (0.00, 2.00) | 0.00  (0.00, 1.00) | 1.00  (1.00, 2.00) | 1.00  (0.00, 3.00) | 0.695 |
| **Ln(FBG/HDL-C),**  **median (IQR)** | 1.40  (1.14, 1.73) | 1.28 (1.12, 1.40) | 1.46 (1.20, 1.75) | 1.26  (1.09, 1.62) | 1.18  (1.03, 1.95) | 1.42  (1.13, 1.83) | 0.221 |
| **Renal disease,**  **n (%)** | 87 (31.0) | 3 (23.1) | 68 (40.2) | 10 (20.4) | 0 (0.0) | 6 (13.3) | <0.001 |
| **Hypertension,**  **n (%)** | 101  (35.9) | 7  (53.8) | 42  (24.9) | 27  (55.1) | 2  (40.0) | 23  (51.1) | <0.001 |
| **Diabetes,**  **n (%)** | 98 (34.9) | 2 (15.4) | 67 (39.6) | 14 (28.6) | 1 (20.0) | 14 (31.1) | 0.269 |
| **IMV, n (%)** | 73 (26.0) | 1 (7.7) | 46 (27.2) | 13 (26.5) | 1 (20.0) | 12 (26.7) | 0.677 |
| **CHF, n (%)** | 191  (68.0) | 6  (46.2) | 169 (100.0) | 7  (14.3) | 4  (80.0) | 5  (11.1) | <0.001 |
| **CPD, n (%)** | 155 (55.2) | 4 (30.8) | 97 (57.4) | 49 (100.0) | 3 (60.0) | 2 (4.4) | <0.001 |
| **90-day mortality,**  **n (%)** | 79 (28.1) | 5 (38.5) | 49 (29.0) | 13 (26.5) | 1 (20.0) | 11 (24.4) | 0.875 |

Data are presented as median (IQR) or n (%). P-values were derived from the Kruskal–Wallis test for continuous variables or the χ² test/Fisher's exact test for categorical variables (when expected cell counts <5). Abbreviations: PH, pulmonary hypertension; SOFA, Sequential Organ Failure Assessment; Ln, natural logarithm; FBG, fasting blood glucose; HDL-C, high-density lipoprotein cholesterol; IMV, invasive mechanical ventilation; CHF, congestive heart failure; CPD, chronic pulmonary disease. Owing to small sample sizes in Group 1 (n=13) and Group 4 (n=5), comparisons involving these groups should be interpreted cautiously due to reduced statistical power and unstable effect estimates.
